# Dynamic nano-triboelectrification using torsional resonance mode atomic force microscopy

**DOI:** 10.1038/srep27874

**Published:** 2016-06-15

**Authors:** Wei Cai, Nan Yao

**Affiliations:** 1 Princeton Institute for Science and Technology of Materials, Princeton University, Princeton, New Jersey, 08544, United States

## Abstract

Understanding the mechanism of charge generation, distribution, and transfer between surfaces is very important for energy harvesting applications based on triboelectric effect. Here, we demonstrate dynamic nanotriboelectrification with torsional resonance (TR) mode atomic force microscopy (AFM). Experiments on rubbing the sample surface using TR mode for the generation of triboelectric charges and *in-situ* characterization of the charge distribution using scanning Kelvin probe microcopy (SKPM) were performed. This method allows the tip to perform lateral oscillation and maintains the tip-sample interaction in the attractive region to ensure high efficiency of the charge generation during the rubbing process. The measured efficiency of generating triboelectric charges can achieve ~10.53 times higher than conventional static/contact mode in the triboelectrification experiments. In addition to the charge generation, local discharging experiments were also performed. This work would provide a new method to generate patterned charges and also be helpful in understanding the mechanism of nanotriboelectrification.

The triboelectric effect demonstrates a great number of novel applications recently in energy harvesting devices^1^,^2^ and self-powered sensors^3^,^4^. Based on this effect, various ambient mechanical energy sources can be collected and converted into electricity which shows good prospects for the development of renewable energy^5^. To harvest the mechanical energy with higher efficiency, it is very important to understand the mechanism of the charge generation, distribution, and transfer between surfaces in this effect. However, due to the complexity of the phenomena, a fundamental understanding about the triboelectrification is rather limited. Budakian and Putterman developed a method that facilitated macroscopic measurements of metal-insulator surfaces in relative motion and they found a quantitative correlation between charging and friction, suggesting that triboelectrification and friction have a common origin^6^,^7^. Experiments using scanning probe microscopy have been reported to investigate the relationship between triboelectrification and friction on the nanoscale^8^,^9^. Various methods were proposed to accurately control the triboelectrification process and quantify contact charging. For example, the surface was firstly rubbed with different types of materials or contacted with micropatterned structures^10^,^11^,^12^,^13^,^14^. Then with the help of electrical AFM (atomic force microscopy) based scanning Kelvin probe microscopy (SKPM), surface charge mapping can be obtained with nanometer resolution^15^,^16^,^17^. But in these techniques, rapidly positioning the probe to the rubbed or contacted region is still a tedious task^13^, because obviously the charges cannot be seen under the optical microscope and as time goes on the charges will decay and diffuse, which causes the distribution to be changed.

Using AFM, the probe itself was used to rub the sample surface, thus the SKPM imaging can be performed *in-situ* after charge generation without the need of searching for the charged surface region^18^,^19^,^20^,^21^,^22^,^23^,^24^. For example, Sun *et al*. used AFM triboelectrification with different applied tip load which is one of the factors affecting the properties of surface charges^21^. In recent years, Zhou *et al*. reported an *in-situ* quantitative method for characterization of the triboelectrification process at the nanoscale^22^ and charge transfer between the surfaces of two materials through a biased electric field^23^. Mirkowska *et al*. studied the electric charging of calcite single crystals by using the tip contact and rubbing methods^24^. These experiments were helpful to understand the fundamental mechanism of the triboelectric process. So far, all AFM based methods mentioned above were using static/contact mode to generate the triboelectric charges. To enhance the surface charge density, what needed is to increase the number of friction cycles^22^. However, since the scan rate is limited in conventional AFM, usually ~1 Hz, the scan process would cost several frames to generate more charges, thus it is time-consuming and less efficient due to charges decay and diffusion.

Compared with the static/contact mode for imaging formation in AFM, dynamic modes including the tapping mode^25^ and the torsional mode^26^,^27^ can provide the tip’s motion in vertical and lateral directions with high frequency during scanning, respectively. Here we show *in-situ* nanotriboelectrification using torsional resonance mode AFM. By rubbing the sample surface with TR (torsional resonance) mode, the triboelectric charges on the surface of insulating material were generated. High-resolution imaging of the triboelectric charge distribution was then performed *in-situ* by SKPM (see schematics in Fig. 1a,b). The experiment results indicate that the efficiency to generate the triboelectric charges using TR mode can achieve ~10.53 times higher compared with the contact mode AFM. Furthermore, we demonstrated *in-situ* manipulation of charging and discharging process on a test sample surface. This work would provide a new method to generate charges with high efficiency and also be helpful in understanding the mechanism of nanotriboelectrification.

## Results and Discussion

Benefited from the capability of tip-based charge generation and *in-situ* charge characterization method, Zhou *et al*. reported the multi-friction effect on triboelectric charge transfer^22^. In the multi-friction effect, as the insulating material’s surface (silicon dioxide) was rubbed by the tip for more frames, the measured surface potential was increased gradually. The amount of charges transferred in each rubbing cycle is related to the difference in the “effective work function” of the probe and the silicon dioxide’s surface^28^. The difference would reduce while more tribocharges accumulated on the rubbed region until the charging reached the saturation state. A phenomenological model was proposed to describe the multi-friction effect. The relationship between the increase rate of the charge density σ and tip rubbing times *n* can be expressed by^22^


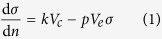


where *V*_*c*_ is a constant depends on the sample material’s property, and *V*_*e*_ is proportional to the amount of existing tribocharges. *k* and *p* are considered as charging efficiency coefficient and charging impedance coefficient, respectively. According to the boundary conditions, 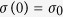 and 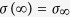. Where, σ_0_ and σ_∞_ are the surface charge density before triboelectric process and the saturate charge density, respectively. The solution of the charge density σ can be obtained by^22^


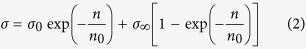


where, *n*_*0*_ = 1/*pV*_*e*_ is the saturation constant and σ_∞_ = *kV*_*c*_/*pV*_*e*_ is the saturate charge density, respectively. From this theory, in unit time, if the surface could be rubbed by the probe for more times, the charge density σ would be higher. The increase rate of the charging would reduce with the increase of the rubbed times and the charge density would approach to saturation. In torsional mode, the AFM tip is vibrating in the lateral direction (parallel to the sample surface). The oscillation-caused multi-friction at each scan point would be helpful to obtain higher charge density during the rubbing process.

**Figure 1:**
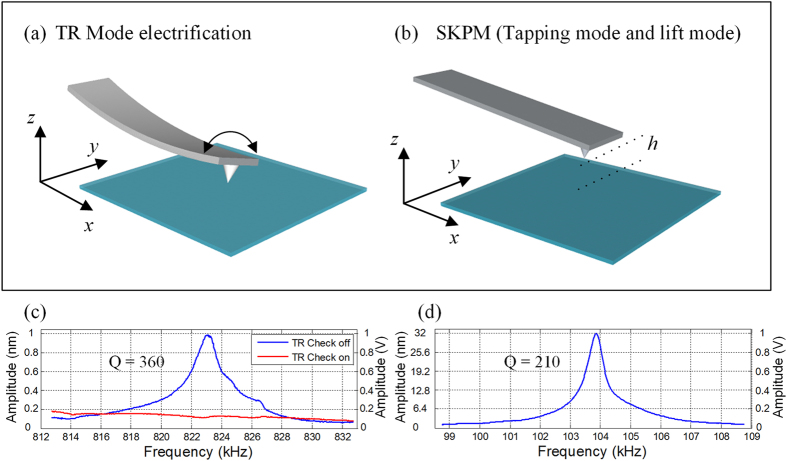
(**a**) Schematic diagram of TR mode for dynamic nanotriboelectrification. (**b**) SKPM mode for characterization of the triboelectric charge distribution. (**c,d**) are the torsional and tapping resonance frequency of the cantilever (blue curves), respectively. As shown in (**c**), TR “check function” in the control program can be used to verify the torsional resonance frequency (red curve).

The TR mode was initially developed to map in-plane mechanical properties, such as friction, shear stiffness, and other tribologically relevant properties and was typically operated in air with the amplitude-modulation scheme^29^,^30^. To set the cantilever working in torsional mode, the first step in experiments is to determine the cantilever’s torsional driving frequency. Before tuning the cantilever, the driving frequency of both tapping and torsion can be pre-evaluated by Euler–Bernoulli beam theory^31^,^32^,^33^ with dimension parameters of the cantilever (see the Supplementary Information). Equation (3) gives the fundamental vertical bending (tapping) frequency *f*_*v,0*_:


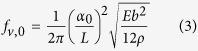


where *α*_*0*_ = 1.875, *L* and *b* are the length and thickness of the cantilever, respectively. *E* is the Young’s modulus of the cantilever material and *ρ* is the density. And equation (4) can be used to determine the fundamental torsional frequency *f*_*t,0*_ of the cantilever^33^,^34^.


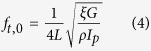


where, *ξ* is a correction coefficient for compensating the warping effect^34^. *I*_*p*_ is the polar moment of inertia. *G* is the shear modulus of the cantilever material. The dimension of the compliant silicon cantilever (see the Methods) we used is 135 μm × 30 μm × 1.5 μm (*L* × *a* × *b*). Young’s modulus of elasticity *E* = 169 GPa, the shear modulus *G* of 50 GPa and a mass density *ρ* of 2330 kg m^−3^ were assumed for the cantilever material^35^. The calculated resonance frequencies are *f*_*v,0*_ = 113 kHz and *f*_*t,0*_ = 843 kHz, respectively. Figure 1c shows the relationship between the torsional oscillation amplitude of the cantilever and its excitation frequency in air (the blue curve). The coupling-check in the control program can be used to compare the flexural bending amplitude (the red curve) with the torsional amplitude to ensure that the cantilever can work in the torsional resonance mode. For the measured fundamental torsional mode, the cantilever has a resonance peak at ~823 kHz with a Q factor of ~360. Figure 1d shows the fundamental flexural bending mode and a resonance peak at ~104 kHz with a Q factor of ~210 is measured. The difference between experimental and theoretical values is mainly due to the dispersion of cantilever’s dimensional parameters, e.g., if the length of the practical cantilever is 2.4% longer than the nominal value, the torsional frequencies of both experimental and theoretical values can match quite well.

**Figure 2:**
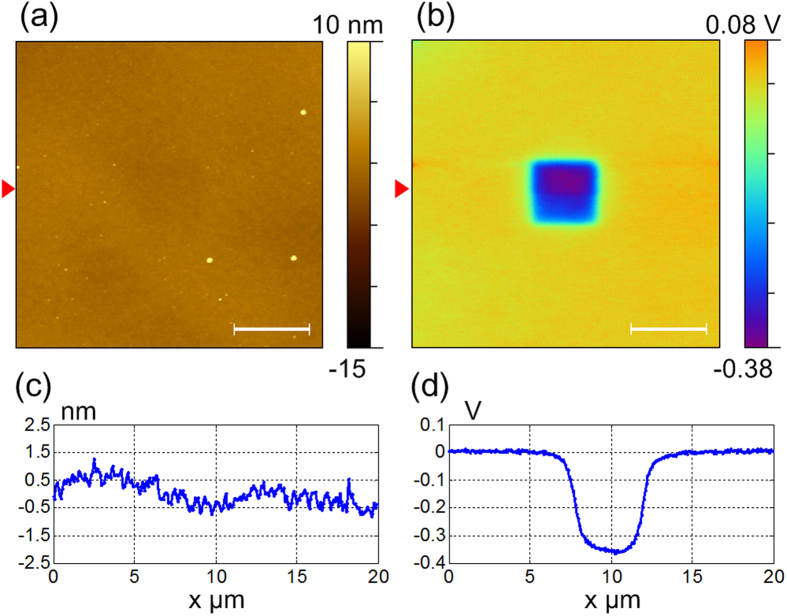
(**a**) Topographic and (**b**) surface potential images of the square region rubbed by TR mode AFM. (**c,d**) are the profile lines extracted from the marked positions in (**a,b**), respectively. The scale bars are 5 μm.

To generate charges on a selected insulating material’s surface, we tried to use the TR mode to rub a square test-region on the surface of a thin silicon dioxide film. No voltage was applied on the metal AFM tip during the rubbing process. In TR mode, once the dithering tip engaged the surface, the torsional resonances would be influenced by the in-plane conservative elasticity of lateral forces as well as dissipative friction forces^36^,^37^. Small amplitude (~300 mV) for TR mode AFM is useful for keeping the soft contact during scanning. This would be ideal for imaging soft samples even in liquid environment^30^. However, small amplitude in rubbing would not generate noticeable charges on the insulating material’s surface based on the charge mapping results in SKPM mode. In order to increase tip–sample interactions during rubbing, the amplitude of torsional free-oscillation was increased to ~3.3 times compared with the ordinary TR mode imaging, and the tip would interact with sample surface aggressively during scanning^33^. The corresponding lateral signal from AFM PSD (position sensitive detector) was ~968 mV in experiments. The torsional sensitivity (~1 nm/V) was calibrated by the method proposed by Huang and Su^38^. So the free oscillation amplitude of the torsional vibration of the tip is ~1 nm at its resonance frequency. This result is in agreement with Yurtsever *et al*. who gave an estimation of the tip’s amplitude in “nm”. They reported that since the twisting angle of the cantilever was very small, the tip oscillated nearly parallel to the surface with typical amplitudes smaller than 1 nm^39^. Before approaching the sample surface, the amplitude feedback set-point ratio was usually at half of the free oscillation. When the tip engaged the surface, the set-point ratio would be adjusted to ~75% automatically. Then, to reduce the wear of the surface and the tip, the set-point ratio was finely adjusted to 90% manually while making sure the tip could still track the surface. The defined rubbing region was typically set to ~4 μm × 4 μm and the scan rate was 1 Hz in experiments. We kept continuous scanning for ~1.5 frames (as in the control program, the scan started from the middle line of the frame). After that, the rubbed region was characterized by SKPM (tapping mode and lift scan, its principle is given in the Supplementary Information). The representative surface topography of the silicon dioxide film and the corresponding surface potential image were shown in Fig. 2a,b. The images have a size of ~20 μm × 20 μm. Topographic image shows a flat surface with several dust particles on it. The surface potential image shows an obvious contrast between the rubbed region and the intact area. Figure 2c,d gives the cross-section lines marked in Fig. 2a,b. From the surface potential profile in Fig. 2d, the rubbed region is ~0.385 V lower than the surrounding area. The sign of the measured potential is negative, which means negative charges were generated and accumulated on this region. This result is in good agreement with that reported by Zhou *et al*.^22^. It also confirms that the silicon is more triboelectrically positive than silicon dioxide, which means that the silicon probe is likely to be positively charged and the silicon dioxide surface is negatively charged correspondingly after the rubbing process. The distribution of accumulated triboelectric charges was illustrated and the corresponding charge densities were proportional to the measured surface potential of SKPM^13^. The density *σ* of charges on the surface of the insulating film can be approximated by the capacitor equation^13^:


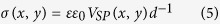


where *V*_*SP*_ is the measured surface potential, *ε* and *ε*_*0*_ are the dielectric constant of the insulating film and the electric permittivity of free space, respectively. The thickness *d* of the insulating layer measured by ellipsometry is ~110 nm. So the calculated density σ is ~−121 μC/m^2^. To rub the surface with large torsional oscillation amplitude (e.g. the free-oscillation amplitude is larger than ~1.1 V and the maximum value on our instrument can achieve ~2 V), self-oscillation can be observed in the height image channel which might be induced by the AFM’s vertical feedback loop. The scan become unstable and the tip cannot track the surface well in this situation.

**Figure 3:**
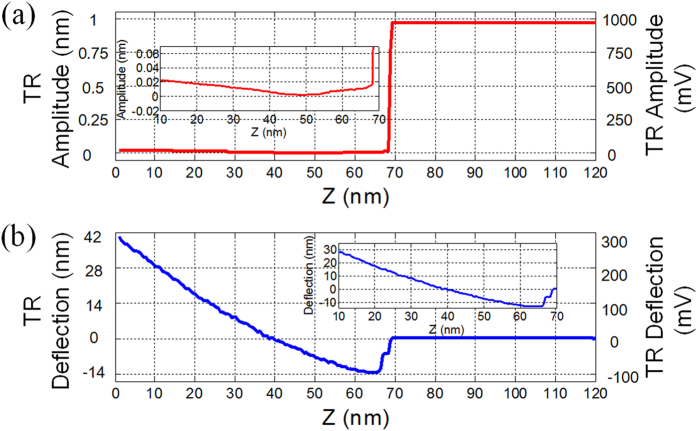
Approaching curves measured in TR mode on a silicon dioxide surface under ambient environment. (**a,b**) are the amplitude and deflection signals versus Z displacement of the tip, respectively. The insets contain the zoomed range in Z displacement from 10 nm to 70 nm.

In order to illustrate tip-sample interactions which have been utilized in TR mode rubbing process, approaching curves were obtained on the same silicon dioxide surface. Figure 3a,b show typical approaching curves of the amplitude and deflection signals of TR mode versus the *Z* displacement of the tip. The tip approached the surface with the parameters (the frequency and the amplitude) that we mentioned above. Firstly, the cantilever amplitude and the deflection signals were not disturbed because there was not enough force applied on the cantilever. Then, at some distance (Z position ~69.8 nm) the amplitude signal dropped drastically from ~968 mV to ~15 mV within ~1.5 nm range. The corresponding deflection signal started to reduce from the same position, which showed attractive forces (usually capillary forces in air) existed between the cantilever and the surface. And the tip jumped into contact with the surface in ~5 nm range (Z position ~64.5 nm). Within this range, the amplitude signal was gradually reduced from ~15 mV to ~10 mV. When the tip approached further, it would cause the increase of the deflection signal because the repulsive force increased, as shown in the inset of Fig. 3b. In the repulsive region, changes of the amplitude signal which first decreased from ~10 mV to ~2 mV then increased to ~26 mV were observed, as shown in the inset of Fig. 3a. During TR mode rubbing, when we regulated the amplitude signal at 90% of free oscillation by the feedback circuit, the tip was operated in the attractive interaction region according to the corresponding deflection signal. In this region, the AFM tip was maintained at low force and continuously had the lateral interaction with the sample surface. It is one of the main characteristics of TR mode AFM that it has the ability to stably maintain the tip in “soft contact” region of the sample surface where the tip is at the boundary between firmly contact and long-range force region^34^. The amplitude of the torsional vibration which helps the triboelectrification to occur between the dithering tip and the sample surface, could be a reflection of friction force^27^. When we used the dynamic friction feedback (the deflection signal for feedback) during rubbing, the tip was operated in the repulsive interaction region according to the approaching curve on the surface. However, because the amplitude signal on the silicon dioxide surface was very small, the measured charges were at the same level as the conventional contact mode (see the Supplementary Information).

**Figure 4:**
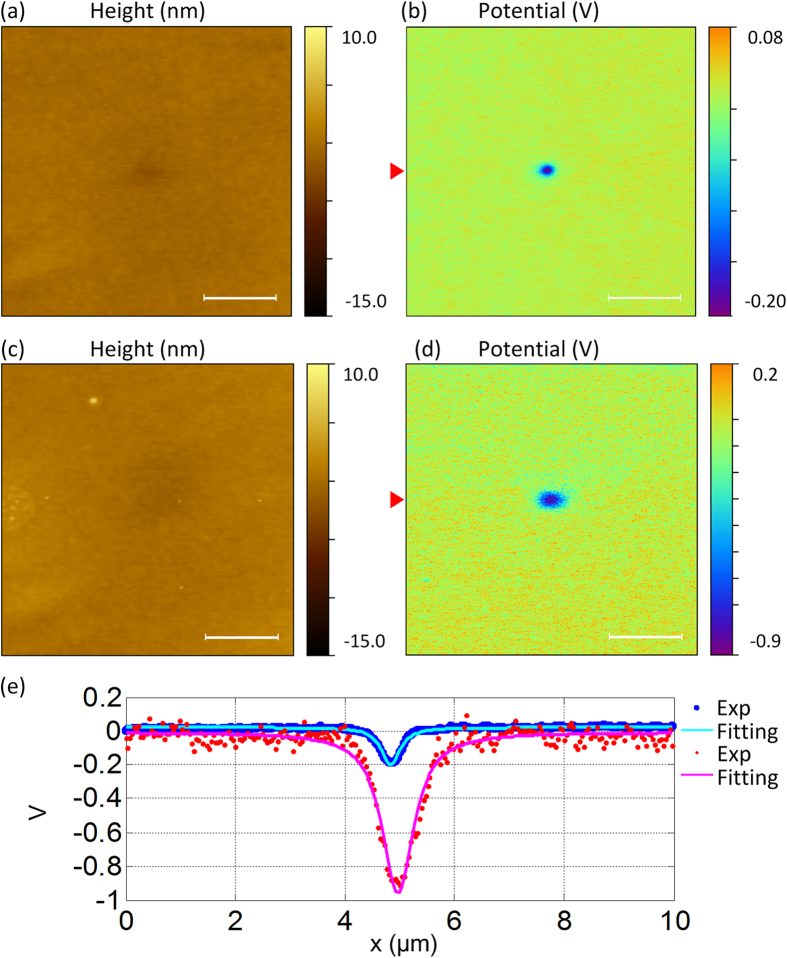
(**a,b**) are the topographic and surface potential images of the silicon dioxide surface after the tip contacted with the surface for ~2 min (engaged with contact mode). (**c,d**) are performed in the same way by TR mode. (**e**) is the profiles extracted from the marked line in (**b,d**). The scale bars are ~2.5 μm.

Figure 4 shows a comparison experiment to generate charges with contact mode and TR mode, respectively. To compare the efficiency of charge generation, the experiment was performed with a single point contact by both modes. So before the tip approached the surface, the scan size was reduced to 0 nm. And after the tip engaged, we kept the tip interacting with the surface for ~2 min and then withdrew. Following that, we used SKPM mode to map the variations of the surface potential. In contact mode, since the scan had been turned off, no friction was applied between the sample and the tip. The amount of generated tribocharges should be very small. However, as shown in Fig. 4a,b, the potential of the contact region is ~0.259 V lower than the surrounding area and the corresponding charge density is ~−81 μC/m^2^. The reason for contact electrification has been discussed by Terris *et al*.^18^. and Sun *et al*.^21^. One explanation is the charge transfer between the tip and the sample surface happened in a contact-separation process^21^. Another possible reason is that the lateral motion of the tip caused friction due to cantilever bending, because usually the cantilever has a small mounting angle (~12°) relative to the horizontal plane^40^. In TR mode, as shown in Fig. 4c,d, the potential of the contact region is ~0.950 V lower than the surrounding area and the corresponding charge density is ~298 μC/m^2^. The surface potential value of the contact region rubbed with TR mode is significantly increased than the contact mode. There are two possible reasons for the enhancement as we mentioned above. One is that torsional oscillation of the cantilever causes the tip to dither laterally while the tip is rubbing the surface. The other is the amplitude feedback can help the tip always work in the attractive region during the rubbing process. Figure 4e shows two line profiles extracted from Fig. 4b,d. Considering that the generated charges’ region is a circular shape and the charge density is uniform within this area, the diameter of the circle can be evaluated by FWHM (the full width at half maximum) of each profile line. By Lorenz fitting, FWHM values are 407.5 nm and 689.4 nm, respectively. So the corresponding areas are 5.217 × 10^5^ nm^2^ and 1.493 × 10^6^ nm^2^. The amount of tribocharges can be evaluated by multiplying the density and the area. Therefore, based on these calculations, the efficiency of using TR mode to generate charges can achieve ~10.53 times higher than the contact mode.

From topographic images, the local surface charging slightly affected the measured AFM topography. So the height of the center region seems lower than the surrounding area, which may cause some errors for topographic tracing in lift mode. However, because the surface of the silicon dioxide film is very flat (the measured height difference was less than 10 nm) and the cross-talk part looks faint, the height difference measured between the rubbed and the intact areas is very small (less than 1 nm). And in SKPM measurement, the lift height we maintained is 50 nm. The small height difference would not cause significant difference in the measured surface potential. Using shorter cantilever may further increase the efficiency, because it has higher torsional resonance frequency. During rubbing, the number of friction cycles at each pixel is higher too. However, in our experiments under the same condition, the measured surface potential level didn’t increase obviously (see the Supplementary Information). One explanation is that the surface charge density reached saturation state at this local area thus the surface potential level would not further increase.

**Figure 5:**
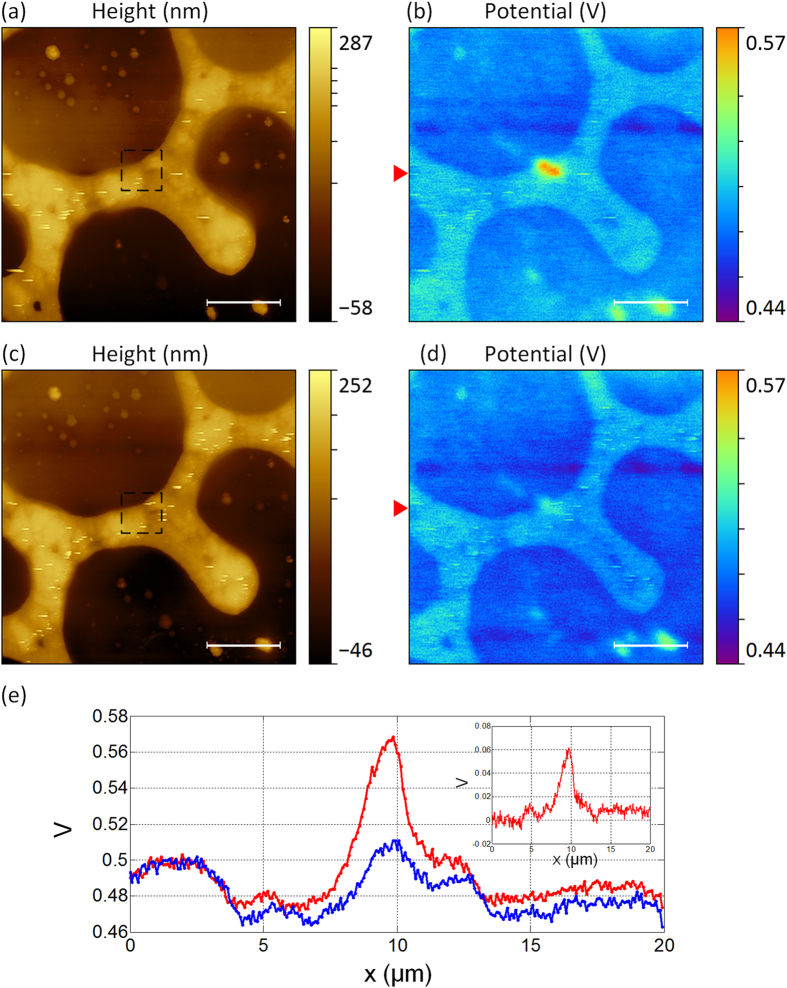
(**a,b**) are the topography and surface potential images of the PS/LDPE test sample. The central region of ~2 μm × 2 μm was rubbed by TR mode. (**c,d**) are the images after discharging process. (**e**) is the profiles extracted from the marked line in (**b,d**). The inset illustrated the potential difference of charging and discharging. The scale bars are ~2.5 μm.

Figure 5 shows an example of charging with TR mode on a specific point of the sample surface and then charges were removed by the same tip. The test sample was a polystyrene (PS) film with phase-separated islands of low-density polyethylene (LDPE) coated on top of a silicon substrate (PS/LDPE, Bruker, US). Charging process was achieved with the method mentioned above to rub the local region of ~2 μm × 2 μm on the test sample surface. After rubbing for two frames, topography and surface potential images were acquired by SKPM, as shown in Fig. 5a,b. The rubbed region has been marked on the figure with dash lines. Based on topography, the higher areas are the LDPE surface and many small islands. The lower areas are the PS surface. This can be further confirmed by phase contrast images, because they have different elastic modulus (see the Supplementary Information). From the surface potential image, different components on the sample surface can also be distinguished with small potential variations of ~0.03 V. The contrast of the surface potential is related to their different amount of surface charges in air^15^. The rubbed region shows ~0.1 V higher than the surrounding area, which indicates the positive charges gathered in the region after rubbing. However, the distribution of the rubbed region is not a perfect square shape. One explanation is that the surface of the PS/LDPE film is not as flat as the silicon dioxide surface and is also much softer, which may affect the charge distribution. Another possible reason is the different surface properties of the PS and LDPE might affect the amount of surface charges^41^. Next, in order to remove tribocharges accumulated on the local area, two frames scan were performed on the same rubbed region using the contact mode with the tip connected to the ground. Then using SKPM mode for potential mapping, the results were shown in Fig. 5c,d. From topographic image, no appreciable wear was observed, which indicates this method would not damage the sample surface. In the corresponding surface potential, most of the gathered charges were removed. But there were still small amount of residual charges found in the rubbed area, which might be caused by transfer process with zero bias during contact electrification^23^. Figure 5(e) gives the potential profiles extracted from Fig. 5b,d which shows the potential difference of ~0.06 V of the rubbed region after charging and discharging, respectively. The inset in Fig. 5e shows the result curve which was obtained by subtracting the after discharging curve (blue) from the charging curve (red). According to “triboelectric series” summarized in the review paper by McCarty and Whitesides, polystyrene (PS) and polyethylene (PE) materials should tend to acquire negative charges. However, in our experiment, the measured sign of tribocharges was reversed. This situation was also found in previous studies. For example, in early works of Terris *et al*., both positive and negative charge regions could be found after the tip contacted the sample surface (PMMA) and the region was substantially larger than the expected contact area^18^. And Sun *et al*. reported that the sign of tribocharges could be reversed by different loaded forces applied on the AFM tip. These studies supported that triboelectrification probably involves more than one mechanism that will affect surface states of the AFM tip and the sample surface. The relative position of the highest occupied level might be changed, which resulted in the reverse of the sign of tribocharges.

In summary, we have presented a new method for dynamic nanotriboelectrification with TR mode AFM which can significantly increase the efficiency to generate the triboelectric charges compared with the contact mode. Experiments about rubbing the sample surface with TR mode for the generation of triboelectric charges and *in-situ* characterization of the charge distribution by SKPM were carried out. This method allows the tip to perform lateral oscillation and keeps the tip-sample interaction in the attractive region by using the amplitude modulation scheme during rubbing, which is especially important for the enhancement of the triboelectric charge generation. In addition, we demonstrated *in-situ* manipulation of the generated charges, i.e. *in-situ* charging and discharging processes were performed on a test sample surface without surface damages. Therefore, this work would help understand the mechanism in nanotriboelectrification and optimize the design of energy harvesting devices and self-powered sensors.

## Methods

The experiments were performed on a commercially available AFM system (Dimension V SPM system with Nanoscope V controller, Bruker, US). A specially designed tip holder (DTRCH, Bruker, US) was employed for working in TR mode. This holder includes dual parallel piezo actuators which can be driven by two sine signals to oscillate the cantilever torsionally or vertically. The excitations of torsional and vertical bending can be switched with the opposite phase and in-phase driven signals, respectively. Working in TR mode, the relative drive voltages applied to the two piezo actuators were adjusted automatically by the software to maximize the RMS (the root mean square) value of the lateral signal from the photodetector. Then, this RMS signal would be used as the amplitude feedback signal during scanning. SKPM (tapping mode) can also be performed with this holder, which allows the *in-situ* measurement of the charge distribution.

For *in-situ* TR mode rubbing and SKPM imaging, a Si-based probe without metal coating just at its tip end was freshly prepared before the experiments^22^. A Pt-Ir coated AFM probe (NSG03/Pt, NT-MDT, Russia) was selected for the tip preparation. It has a flexural resonance frequency of ~90 kHz, a nominal spring constant of ~1.74 N/m, and a radius of curvature of ~35 nm. Typically, this probe has the fundamental torsional frequency of ~850 kHz. The metal coating was slightly worn at the tip ending by contact mode scanning on a clean glass substrate with a normal force of ~100 nN for ~20 minutes. The preset scan range and scan rate are ~2 μm and 1 Hz, respectively.

Our charge patterning procedures were inspired by Zhou *et al*.^22^. Firstly, the AFM was operated in TR mode to scan a silicon dioxide surface for several frames. Secondly, we switched to SKPM mode to measure the topography and distribution of the accumulated tribocharges. In the control software, the tip had to be withdrawn before changing to SKPM mode. In order to relocate the tip within the same area once you engage again, we carefully lowered the safety clearance in the engage setting to less than ~50 μm. So once withdrawn and switched the working mode, the tip would not lift very far away from the sample surface. And we also turned on the close-loop control of the scanner during rubbing and scanning for drift compensation. The thickness of the silicon dioxide layer was determined by a spectroscopic ellipsometer (M-2000, JA Woollam, US).

SKPM is a two-pass scan mode on our AFM. On the first pass, the cantilever was vibrated near its flexural resonant frequency *f*_*0*_ (tapping mode) by the piezo components and the sample topography was measured. Then, on the second pass, surface potential imaging was realized under the lift-mode with a pre-defined lift height Δ*Z* = 50 nm. During lift scanning, the piezo components that normally vibrate the cantilever were turned off. An AC voltage *V*_*ac*_and a DC voltage were applied on the conductive cantilever. The AC voltage was used to drive the cantilever to vibrate in electrical way because of electrostatic force interactions between the tip and the sample surface. The AC frequency is set to be ~2 kHz lower than the cantilever resonance frequency (~120 kHz) and the amplitude is ~560 mV. As a potential signal source, the DC voltage is used to track the surface potential at each pixel. The scan rate is 0.75 Hz in SKPM imaging and the silicon dioxide sample’s back is grounded to diminish the background noise. All of the experiments were performed in air under ambient laboratory conditions. The relative humidity within the laboratory environment was maintained the same (~35%) by the central air-conditioning automatically. AFM images were processed using the Gwyddion software (version 2.40).

## Additional Information

**How to cite this article**: Cai, W. and Yao, N. Dynamic nano-triboelectrification using torsional resonance mode atomic force microscopy. *Sci. Rep*. **6**, 27874; doi: 10.1038/srep27874 (2016).

## Supplementary Material

Supplementary Information
