# Supplementary material for: Dynamic nano-triboelectrification using torsional resonance mode atomic force microscopy
Source: Sci Rep. 2016 Jun 15;6:27874. doi: 10.1038/srep27874 (PMC4908601; doi:10.1038/srep27874)
Supplement: Supplementary Information [file srep27874-s1.doc]

## Supplementary information

Dynamic nano-triboelectrification using torsional resonance mode atomic force microscopy

*Wei Cai and Nan Yao^*^*

Princeton Institute for Science and Technology of Materials, Princeton University, Princeton, New Jersey, 08544, United States

_____________________________

*Author to whom correspondence should be addressed. Electronic mail: nyao@princeton.edu.

## 1. The tapping and torsional driving frequency

The driving frequency of both tapping and torsional mode can be pre-evaluated by Euler–Bernoulli beam theory with dimension parameters of the cantilever. For tapping (vertical bending) mode, suppose *x* is the direction along the cantilever and *y* is the vertical direction. The equation of the motion of the cantilever can be expressed by:

$$EI\frac{\partial^{4}y}{\partial x^{4}}\text{+}\rho A\frac{\partial^{2}y}{\partial t^{2}}=0$$

Where, *I* is the area moment of inertial which can be obtained by$I=ab^{3}/12$. *a, b* are the width and thickness of the cantilever, respectively. *E* is the Young’s modulus of the cantilever material and *ρ* is the density. *A=ab* is the rectangle cross-section area. For the clamp-free cantilever, the general solution is:

$$y(x,t)=y(x)\text{[}cos(\omega_{n}t+\theta)\text{]}$$

Where *n* is the order of the mode. Inserting Eq. into Eq., we can obtain the angular frequency *ω_n_*:

$$\omega_{n}=k_{n}^{2}\sqrt{\frac{EI}{\rho A}}$$

Boundary conditions[^1^](#_ENREF_1) for the motion of the clamp-free cantilever are used to calculate *k_n_* which must meet a simple implicit equation:

$$\cos k_{n}L\cosh k_{n}L+1=0$$

The first value *k_0_* is approximately equal to *α_0_/L*, where *α_0_≈*1.875. So, the fundamental vertical bending frequency *f_v,0_*  is:

$$f_{v,0}=\frac{1}{2\pi}\left(\frac{\alpha_{0}}{L}\right)^{2}\sqrt{\frac{E\text{I}}{\rho A}}=\frac{1}{2\pi}\left(\frac{\alpha_{0}}{L}\right)^{2}\sqrt{\frac{Eb^{2}}{12\rho}}$$

For torsional mode, the equation of the cantilever’s motion can be expressed by[^3^](#_ENREF_3):

$$c_{T}\frac{\partial^{2}\theta}{\partial x^{2}}=\rho J\frac{\partial^{2}\theta}{\partial t^{2}}+\gamma_{air}\frac{\partial\theta}{\partial t}$$

Where *θ* is the cantilever rotation and *c_T_ = ab^3^G/3* is the torsional stiffness and *J* = *(a^3^b + ab^3^)/12* is the area moment of inertial about the torsional axis. The terms *a*, *b*, *L* and *ρ* represent cantilever width, thickness, length and density, respectively. The term *γ_air_* is the damping coefficient of air.[^3^](#_ENREF_3) Air damping does not shift the resonant frequency of a free mode significantly, allowing one to calculate the resonant frequencies of the *n*th free mode without damping. In the similar way, we can obtain the following equation:[^4^](#_ENREF_4)

$$f_{T,n}=\frac{2n-1}{4L}\sqrt{\frac{c_{T}}{\rho J}}$$

To correct the warping effect caused by the tip’s mass, the warping factor is induced and shown as:[^3^](#_ENREF_3)

$$\xi\text{=}\frac{1}{3}ab^{3}(1-0.632\frac{b}{a})$$

So fundamental torsional frequency *f_t,0_* of the cantilever can be determined by:

$$f_{t,0}=\frac{1}{4L}\sqrt{\frac{\xi G}{\rho J}}$$

## 2 Scanning Kelvin probe microscopy

Scanning Kelvin probe microscopy (SKPM) or Kelvin probe force microscopy (KPFM) is a AFM-based method to measure the local contact potential difference between a conductive tip and a sample.[^5^](#_ENREF_5) It has the capability to map electrostatic potential variations on the sample surface.[^6^](#_ENREF_6) The principle of SKPM can be summarized by nullifying the electrostatic force acted on the cantilever. By applying an AC voltage (*V_AC_*) and a DC voltage (*V_DC_*) to the AFM conductive tip, *V_AC_* generates oscillating electrical forces between the AFM tip and sample surface, and *V_DC_* nullifies the oscillating electrical forces that originated from *V_CPD_* (contact potential difference) between the tip and sample surface. In insulators, the surface potential depends mostly on fixed charges at the sample surface.[^6^](#_ENREF_6) The electrostatic force between the AFM tip and the sample is given by:[^5^](#_ENREF_5)

$$F(z)=-\frac{1}{2}\Delta V^{2}\frac{dC(\mathrm{z})}{dz}$$

where *z* is the direction normal to the sample surface, *ΔV* is the potential difference between *V_CPD_* and the voltage applied to the AFM tip, and *dC/dz* is the gradient of the capacitance between the tip and the sample surface. When *V_AC_* sin(ωt) + *V_DC_* is applied, the voltage difference *ΔV* will be:

$$\Delta V=V_{tip}-V_{CPD}=(V_{DC}\text{-}V_{CPD})+V_{AC}\sin(\omega\mathrm{t})$$

The corresponding electrostatic force can be rewritten as:

$$F(z)=-\frac{1}{2}\frac{dC(\mathrm{z})}{dz}[(V_{DC}\text{-}V_{CPD})+V_{AC}\sin(\omega\mathrm{t})]^{2}$$

By using the lock-in technique, the force component *F_ω_* with frequency *ω* is extracted from the electrostatic force between the tip and the surface:

$$F_{\omega}=-\frac{dC(\mathrm{z})}{dz}(V_{DC}\text{-}V_{CPD})V_{AC}\sin(\omega\mathrm{t})$$

Where *V_DC_* can be regulated at each pixel during imaging, if *V_DC_= V_CPD_*, the force *F_ω_* would be nullified. Subsequently, the oscillation amplitude of the cantilever would be reduced to 0 V. The value of *V_DC_* is acquired for each point on the sample surface, composing a map of the surface potential of the scanned area.

## 3 TR mode with dynamic friction feedback and shorter cantilevers

We also performed torsional mode AFM with dynamic friction feedback. In this mode, the feedback signal is the deflection of the cantilever. So the tip is pressed onto the surface with torsional oscillation. According to the approaching curve on the surface, the amplitude signal on the silicon dioxide surface is very small. So this mode is very similar to the contact mode. The surface potential image of the rubbed region is shown as Figure S1 (a). The image has a size of 20 μm ×10 μm. The rubbed region is ~0.139 V lower than surrounding area, which is at the same level as the contact mode.


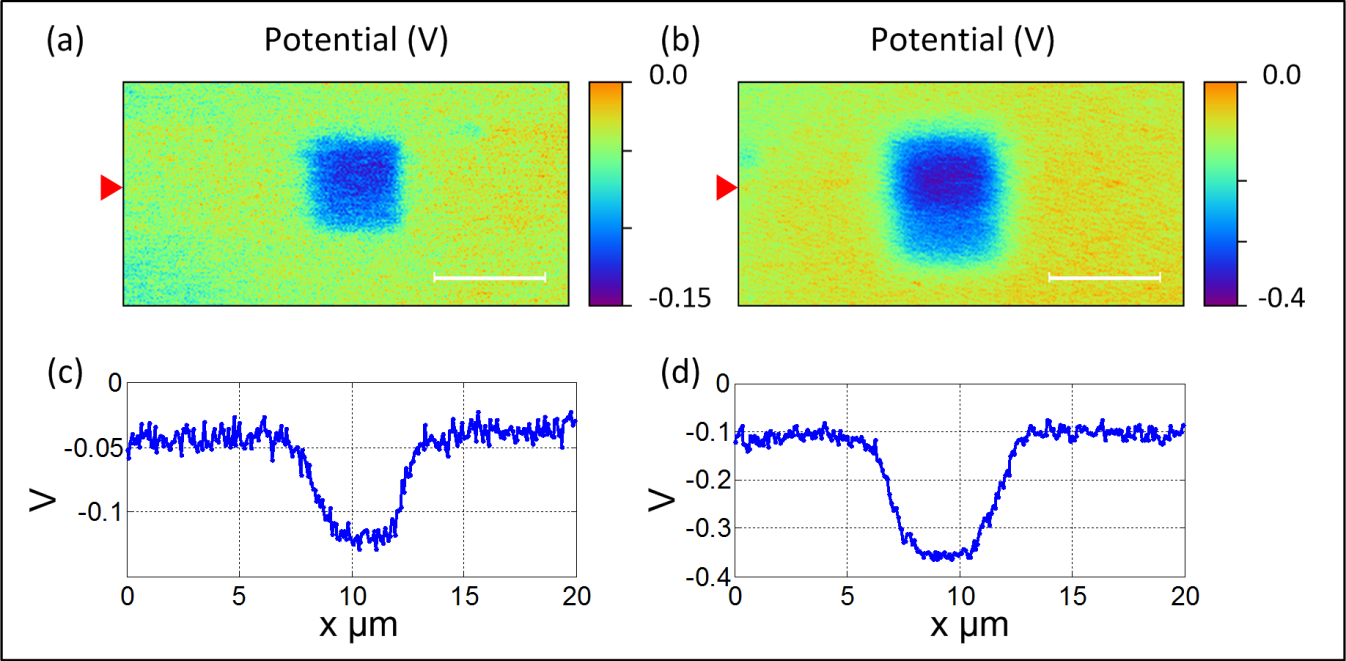


Figure S1. (a) and (b) show the triboelectric charges on the surface after TR mode with dynamic friction feedback and a NSG10/Pt cantilever, respectively. The scale bar is ~5 µm in each image. (c) and (d) give the cross-section lines marked in Figure S1 (a) and (b).

We also performed TR mode with shorter cantilever (NSG10/Pt, NT-MDT, Russia), of which the torsional resonance frequency *f_t,0_*  is ~2 times higher (~1.637 MHz in our experiments). The surface potential image of the rubbed region is shown as Figure S1 (b). The image has a size of 20 μm ×10 μm. From the surface potential profile in Figure S1 (d), the rubbed region is ~0.374 V lower than surrounding area. The results show the measured surface potential level didn't increase obviously by using the shorter cantilever. One explanation is that the surface charge density reached saturation state at this local area thus the surface potential level would not further increase.

## 4 AFM Phase image of the test sample


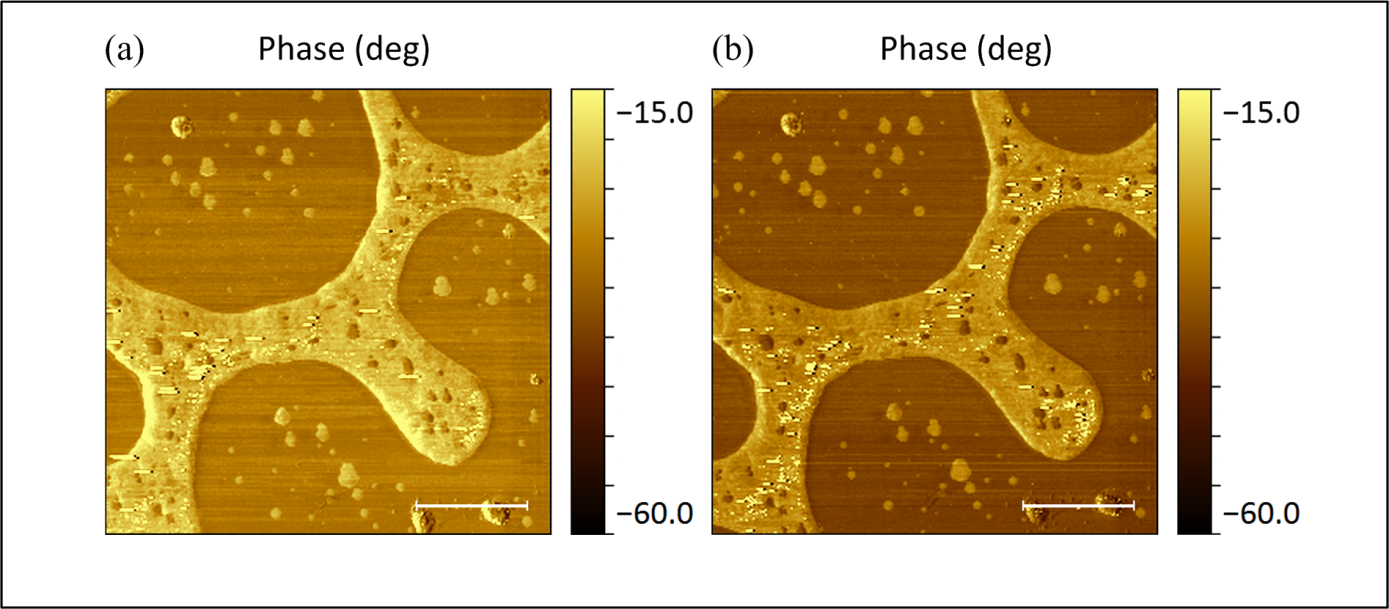


Figure S2. The phase image of the test sample (PS/LDPE) shows different components on the surface. (a) and (b) are phase images after charging and discharging, respectively. The scale bar represents ~5 µm.

Phase images of the test sample (PS/LDPE) after charging and discharging experiments are shown in figures S2. Using the AFM phase contrast, different components on the sample surface can be distinguished because a stiffer material (such as PS with elastic moduli in the 2-3 GPa range) typically shows a darker contrast compared to the softer one (such as LDPE with elastic moduli in the 150-290 MPa range).[^7^](#_ENREF_7) Combining the topographic image with the phase image, the higher areas are the LDPE surface and a lot of small islands while the lower areas are the PS surface.

## References

1 Sarid, D. *Scanning force microscopy: with applications to electric, magnetic, and atomic forces*. (Oxford University Press, 1994).

2 Rabe, U., Janser, K. & Arnold, W. Vibrations of free and surface-coupled atomic force microscope cantilevers: Theory and experiment. *Rev Sci Instrum* **67**, 3281-3293(1996). doi:10.1063/1.1147409

3 Su, C., Huang, L., Prater, C. B. & Bhushan, B. Torsional resonance microscopy and its applications in *Applied Scanning Probe Methods V* 113-148 (Springer, 2007). doi:10.1007/978-3-540-37316-2_6

4 Reinstädtler, M., Kasai, T., Rabe, U., Bhushan, B. & Arnold, W. Imaging and measurement of elasticity and friction using the TRmode. *J Phys D Appl Phys* **38**, R269(2005). doi:10.1088/0022-3727/38/18/R01

5 Melitz, W., Shen, J., Kummel, A. C. & Lee, S. Kelvin probe force microscopy and its application. *Surf Sci Rep* **66**, 1-27(2011). doi:10.1016/j.surfrep.2010.10.001

6 Rezende, C., Gouveia, R., Da Silva, M. & Galembeck, F. Detection of charge distributions in insulator surfaces. *J Phys Condens Matter* **21**, 263002(2009). doi:10.1088/0953-8984/21/26/263002

7 Magonov, S. Exploring Nanomechanical Properties of Materials with Atomic Force Microscopy. *NT-MDT Application Note 085*(2012).
